# Supplementary material for: MetaRibo-Seq measures translation in microbiomes
Source: Nat Commun. 2020 Jun 29;11:3268. doi: 10.1038/s41467-020-17081-z (PMC7324362; doi:10.1038/s41467-020-17081-z)
Supplement: Supplementary file 10 — Supplementary Data 7 [file 41467_2020_17081_MOESM10_ESM.zip › File2/Confidence_High_Taxonomy/96136_out.krona.html]

Javascript must be enabled to view this page.

members
magnitude
magnitudeUnassigned
count
unassigned
taxon
rank

96136\_out

2

superkingdom
2
2

2
phylum
976

200643
2
class

171549
order
2

family
2
171552

genus
1
838

species
1

SRS097867\_contig\_number\_4479
60133

1852368
1

SRS064014\_contig\_number\_11617
species
